# Resistive and reactive changes to the impedance of intracortical microelectrodes can be mitigated with polyethylene glycol under acute *in vitro* and *in vivo* settings

**DOI:** 10.3389/fneng.2014.00033

**Published:** 2014-08-04

**Authors:** Salah Sommakia, Janak Gaire, Jenna L. Rickus, Kevin J. Otto

**Affiliations:** ^1^Weldon School of Biomedical Engineering, Purdue UniversityWest Lafayette, IN, USA; ^2^Physiological Sensing Facility at the Bindley Bioscience Center and Birck Nanotechnology Center, Purdue UniversityWest Lafayette, IN, USA; ^3^Department of Biological Sciences, Purdue UniversityWest Lafayette, IN, USA; ^4^Department of Agricultural and Biological Engineering, Purdue UniversityWest Lafayette, IN, USA

**Keywords:** intracortical microelectrodes, foreign-body reaction, impedance spectroscopy, polyethylene glycols, dip coating

## Abstract

The reactive response of brain tissue to implantable intracortical microelectrodes is thought to negatively affect their recordable signal quality and impedance, resulting in unreliable longitudinal performance. The relationship between the progression of the reactive tissue into a glial scar and the decline in device performance is unclear. We show that exposure to a model protein solution *in vitro* and acute implantation result in both resistive and capacitive changes to electrode impedance, rather than purely resistive changes. We also show that applying 4000 MW polyethylene glycol (PEG) prevents impedance increases *in vitro*, and reduces the percent change in impedance *in vivo* following implantation. Our results highlight the importance of considering the contributions of non-cellular components to the decline in neural microelectrode performance, and present a proof of concept for using a simple dip-coated PEG film to modulate changes in microelectrode impedance.

## Introduction

Failure of intracortical microelectrodes typically manifests as increased impedance and decreased signal to noise ratio, and is thought to be associated with the formation of a dense glial scar and loss of neuronal density. *In vivo* impedance monitoring is a common tool to assess the functionality of implanted intracortical microelectrodes, and has been used to infer the progression of the reactive tissue response to implanted intracortical microelectrodes (Williams et al., 1999, 2007; Vetter et al., 2004). Recent research shows that changes in electrical properties monitored by impedance spectroscopy do not always perfectly correlate with cellular responses (Prasad et al., 2012; Prasad and Sanchez, 2012), implicating additional biotic and abiotic factors. *In vitro* testing in 3D gel constructs reveal that different glial cells adhered to the surface of a microelectrode have different impedance profiles (Frampton et al., 2010). One factor that has not been well investigated is the adsorption of proteins and other biomolecules. While adsorbed proteins have been implicated in the biological response (Leung et al., 2008), their effects on the electrical impedance of intracortical microelectrodes have not been previously described with impedance spectroscopy. Prevalent electrical circuit models of the tissue electrode interface assume that adsorbed proteins result in purely resistive impedance changes (Johnson et al., 2005; Otto et al., 2006; Williams et al., 2007), but there is not sufficient empirical verification of this assumption. To the best of the authors’ knowledge, there are no reports in the literature on the effects of adsorbed proteins or non-cellular components on the impedance of intracortical microelectrodes.

Another aspect to the problem of biomolecule adsorption is the question of preventing detrimental changes to the electrical characteristics of intracortical microelectrodes using simple and cost effective approaches. For implantable devices in other biological systems, protein-resistant or anti-fouling treatments are commonplace (Salacinski et al., 2001; Bluestein et al., 2010; Li and Henry, 2011). One of the most common materials used to enhance the biocompatibility of biomedical implants is polyethylene glycol (PEG). Due to its hydrophilic nature, PEG prevents the adsorption of proteins by reducing access to the more hydrophobic surface onto which proteins prefer to bind (Michel et al., 2005). Typically, PEG is chemically grafted onto a substrate and reliably reduces protein adsorption (Sharma et al., 2004a,b; Muthusubramaniam et al., 2011). In the context of intracortical microelectrodes, PEG has traditionally been used as a scaffold for thick drug eluting hydrogels (Winter et al., 2007; Rao et al., 2012), the size scale of which might exacerbate neuronal displacement. Thinner conformal microgel coatings which incorporate PEG as a cross linker have been investigated, but do not significantly improve the chronic tissue response (Gutowski et al., 2014). Free-floating PEG injected intravenously has been reported to improve cellular and behavioral recovery following traumatic brain injury (Koob et al., 2005, 2008; Koob and Borgens, 2006). Because of the complexity of the reactive tissue response of the brain to implanted microelectrodes, and the fact that the cause of the majority of chronic microelectrode failures are unknown (Barrese et al., 2013), the long-term effects of a simply applied anti-fouling coating on the tissue response cannot be confidently predicted. In the short-term, however, it is possible that such a simply applied anti-fouling coating might improve the electrical properties of acutely implanted neural microelectrodes, which, in turn, might improve the accuracy of impedance monitoring as a predictive tool for the progression of the tissue response. The authors are not aware of any reports in the literature that examine the effects of a simple dip-coated PEG film on the electrical properties of neural microelectrodes under acute *in vitro* or *in vivo* settings.

The primary objective of this paper is to quantify the acute effects on microelectrode impedance of adsorbed proteins *in vitro*, and non-cellular molecular components *in vivo*. A secondary objective is to present a proof of concept on the use of an aqueous dip-coated PEG film in preventing impedance changes to neural microelectrodes during acute timescales. For this proof of concept experiment, a molecular weight of 4000 Da was chosen according to literature reports demonstrating optimal antifouling properties at this molecular weight (Su et al., 2009). We first present an analysis of changes in intracortical microelectrode impedance following immersion in a model protein solution mimicking *in vivo* brain protein concentration. Total impedance, resistance, and reactance are analyzed at different frequency values to quantify the contribution of adsorbed proteins to the impedance changes affecting electrode performance. We show that a dip-coated film of a relatively high molecular weight PEG prevents changes in impedance upon immersion in protein solution. We then demonstrate in an acute *in vivo* experiment that increases in microelectrode impedance after insertion into the cortex can be reduced by applying the same PEG treatment to the electrode shank and implantation site.

## Materials and methods

### *In vitro* study with model protein solution

Electrochemical measurements of 16-channel single shank Michigan electrode arrays (CNCT, Ann Arbor, MI) were made using an Autolab potentiostat PG-STAT12 with a built-in frequency response analyzer (EcoChemie, Utrecht, The Netherlands). For this study, a three-electrode cell configuration was used with the microelectrode site functioning as the working electrode, a large-area Pt wire functioning as the counter electrode, and an Accumet, gel-filled, KCl saturated calomel electrode (Thermo Fischer Scientific, Fair Lawn, NJ) functioning as the reference electrode. This three-electrode setup was chosen for this study because of its ability to isolate the electrode/solution interface impedance component.

For each electrode array, cyclic voltammetry (CV) was performed by sweeping the applied voltage from −0.6 to +0.8 V at a scanning rate of 1 V/s to determine sites with broken or poor connections, designated as those sites exhibiting a maximum current below 1 nA. These sites were discarded from the analysis, thus yielding a total of 30 functional sites on three different electrode arrays.

Electrochemical impedance spectroscopy (EIS), using the PGSTAT12, was used to measure the impedance of the electrode sites with the application of 15 sequentially applied sinusoidal waves at logarithmically spaced frequencies ranging from 46 Hz to 10 kHz, with an amplitude of 25 mVRMS. For each electrode, impedance spectroscopy was performed in PBS following each of three different treatments: (a) no treatment; (b) immersion in a 10% solution of bovine serum albumin (BSA) (Sigma-Aldrich, St. Louis, MO) in PBS, the concentration of which was chosen to mimic protein concentration in rat cerebral cortex (Banay-Schwartz et al., 1992); and (c) immersion in a 20% solution of 4000 MW PEG (Alfa-Aesar, Ward Hill, MA), air-drying for 1 min, then immersion in BSA. Immersion and subsequent removal from the described solutions was done at a controlled velocity using a micro-manipulator. An additional volume of 100 μl of PEG was further applied directly onto the microelectrode shank as it was immersed in BSA to mimic a topical application. Electrodes were rinsed with deionized water and anodically cleaned between the different treatments using 10-s long DC pulses, as described previously (Sommakia et al., 2009). Bode and Nyquist plots were generated for all treatment groups. Comparisons of the resistance, reactance and total impedance were done at 50 Hz, 100 Hz, 1 kHz, and 10 kHz.

### Acute *in vivo* study

The laboratory animal protocol for this work was approved through the Purdue Animal Care and Use Committee (West Lafayette, IN, USA), and conforms to the guidelines of the US National Institutes of Health. Three Sprague Dawley rats (Harlan Laboratories, Indianapolis, IN) were used for this study. Each rat was anesthetized with 2% isofluorane, then transferred to a stereotactic frame and maintained with 0.5–1% isofluorane delivered through a nose cone. The head was shaved and swabbed with alternating washes of betadine and alcohol, and an eye lubricating ointment applied. Lidocaine was injected subcutaneously at multiple positions in the head, and then a midline incision approximately 2 cm long was made along the cranium with a scalpel. The underlying pericranium was removed to expose the skull. A single burr hole was made with a dental drill towards the back of the head, slightly anterior to the ears, and a stainless steel bone screw attached to a segment of platinum wire was threaded into it to serve as a counter electrode. Bilateral craniotomies about 2.5 mm in diameter were made with a dental drill, approximately 2.5 mm anterior to Bregma and 2 mm lateral to the midline. For each craniotomy, a slit was made in the dura using surgical microscissors. One craniotomy serving as a control was wetted with 0.9% sterile saline before electrode insertion, while the other craniotomy was wetted with 20% w/v solution of 4000 MW PEG in MilliQ water prior to electrode insertion.

16-channel single-shank Michigan probes were also used for this study. Because of the difficulty of achieving a three-electrode setup in a surgical setting, a two-electrode setup was chosen instead. Prior to insertion into the cortex, baseline impedance was established *in vitro* by performing the same EIS procedure described above, but in a two-electrode setup. The electrode was then manually inserted into the exposed cortex of the control craniotomy using a magnet-stabilized micromanipulator, and EIS was measured 5 min after insertion. The electrode was then removed, rinsed with MilliQ water, and cleaned by applying a DC bias of 1.5 V for 10 s in PBS, as described previously (Sommakia et al., 2009). After verifying the return of the 1 kHz impedance to baseline, the electrode was dip-coated at a controlled velocity using a micromanipulator in a 20% solution of 4000 MW PEG in MilliQ water for 1 min and allowed to dry for 2 min. The PEG-coated electrode was then inserted using the micromanipulator into the other craniotomy wetted with PEG, and EIS was performed again.

### Statistical analysis

Statistical analysis was performed using the SAS 9.3 statistical package (SAS Institute, Cary, NC). A general linear model (GLM) procedure was used to perform a one way ANOVA with block to remove variations between the different electrodes by treating electrodes as a statistical block. Tukey *post-hoc* tests were used to identify statistically significant differences between the groups at a significance level of *α* = 0.05. Plots were generated using MATLAB (The MathWorks Inc., Natick, MA).

## Results

### Analysis of impedance changes with model protein solution and PEG *in vitro*

**Figure 1:**
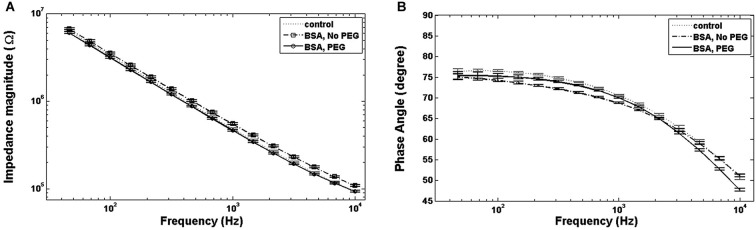
**Bode plots. (A)** For electrodes immersed in BSA with no PEG coating, the total impedance magnitude plot is higher than the control, while electrodes coated with PEG prior to BSA immersion show an impedance magnitude plot indistinguishable from control. **(B)** Electrodes immersed in BSA with no PEG coating show lower phase angles at lower and intermediate frequencies compared to the control, while the phase angle for electrodes coated with PEG prior to immersion in BSA exhibit smaller phase angles at all frequencies.

**Figure 2:**
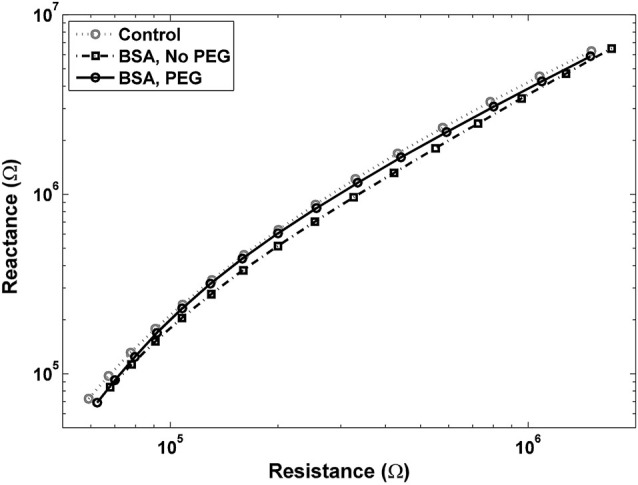
**Nyquist plot for electrodes pretreated with PEG prior to immersion in BSA is close to the control plot, with a slight shift to the left at lower frequencies, indicating a decrease in resistance**. Nyquist plot for electrodes not pretreated with PEG before BSA shows a shift up and to the right, indicating increases in both resistance and reactance.

An examination of Bode plots reveals that the gain for electrodes immersed into is higher than for uncoated controls for all frequencies, indicating an overall increase in impedance. Electrodes treated with PEG prior to BSA immersion show more congruence with the gain of uncoated electrodes (Figure 1A). The phase for non-PEG treated electrodes immersed in BSA exhibits lower angles at the lower end of the frequency spectrum, and the difference is most pronounced in the middle of the spectrum between 200–500 Hz (Figure 1B). In Figure 2, the Nyquist plot for non-PEG treated electrodes immersed in BSA reveal a shift up and the right compared to the controls, indicating both increased resistance and reactance, with the most pronounced divergence occurring toward the middle of the plot. In contrast, the plot for electrodes pretreated with PEG shows more congruence with uncoated controls in the middle of the plot and slight divergence at the lower end of the plot, corresponding to the higher frequencies. To better understand the details of these changes and their potential implication for mitigation attempts, we examined the percent changes in resistance, reactance, and total impedance at various points across the frequency spectrum.

**Figure 3:**
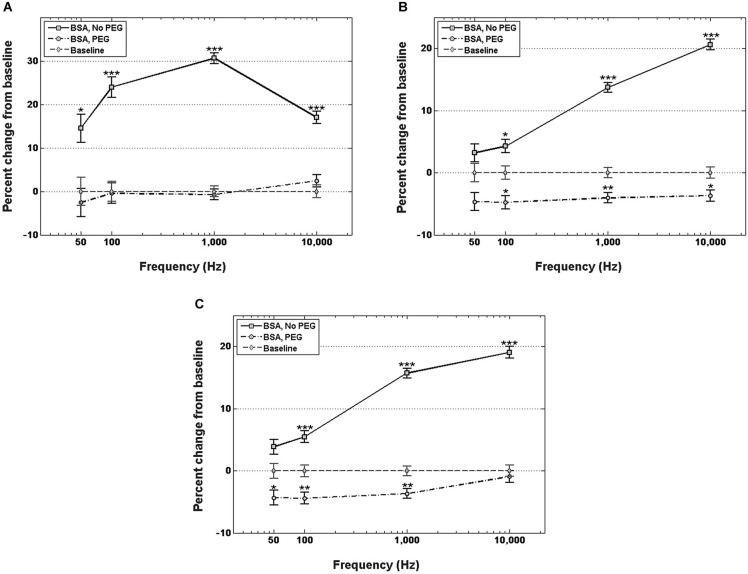
**Changes in electrode impedance following immersion in BSA, without and with PEG treatment. (A)** Resistance of electrodes immersed in BSA with no PEG treatment exhibits significant increases compared to control at all observed frequencies, notably an increase in resistance of 30.7% compared to control at 1 kHz. Electrodes treated with PEG prior to immersion in BSA exhibit no significant changes in resistance compared to control. **(B)** Reactance of electrodes immersed in BSA with no PEG treatment exhibit significant increases at frequencies greater than 50 Hz, with the highest increase observed at 10 kHz. PEG treatment prior to immersion in BSA resulted in minor, but significant decreases at frequencies greater than 50 Hz. **(C)** Changes in total impedance closely match changes in reactance. Error bars represent the standard error of the means. Single asterisks (*) respresent *p* < 0.05, double asterisks (**) represent *p* < 0.001, triple asterisks (***) represent *p* < 0.0001.

Figure 3A shows percent changes in the real component of the impedance, i.e., resistance, relative to the control at four frequency values across the spectrum. Electrodes immersed in BSA without PEG pretreatment exhibited statistically significant increases in the resistance compared to the uncoated control at examined frequencies. The highest resistance increase relative to control was in the middle of the frequency spectrum, specifically at 1 kHz, with a 30.7% increase in resistance. At 50 Hz, the increase in resistance for the BSA coated electrodes without PEG pretreatment was 14.5%, at 100 Hz, the resistance increase was 23.9%, and 10 kHz, the resistance increase was 17%. The electrodes pretreated with PEG prior to BSA immersion, on the other hand, did not exhibit any significant differences in resistance compared to the uncoated controls at all examined frequencies.

Figure 3B shows percent changes in the imaginary component of the impedance, i.e., reactance, relative to the control at four frequency values. For electrodes not pretreated with PEG prior to immersion in BSA, no significant increase in reactance was observed at 50 Hz or 100 Hz, while increases of 12% and 15% were observed at 1 kHz and 10 kHz, respectively. In contrast, electrodes pretreated with PEG prior to immersion in BSA exhibited modest decreases in the reactance relative to control at all frequencies (−6.3% at 50 Hz, −6.3% at 100 Hz, −4.2 at 1 kHz, and −4.8% at 10 kHz). Figure 3C shows the percent changes in total impedance relative to the control at four frequency values. For electrodes not pretreated with PEG prior to BSA immersion, no significant difference in total impedance was observed at 50 Hz, but progressive increases in the impedance were observed at the higher frequencies (4.5% at 100 Hz, 13.5% at 1 kHz, 15.3% at 10 kHz). For electrodes pretreated with PEG prior to BSA immersion, modest decreases in total impedance were observed at the lower frequencies (−5.8% at 50 Hz, −5.6% at 100 Hz, −3.9 at 1 kHz), while no significant difference in the total impedance compared to the control was observed 10 kHz.

### Analysis of impedance changes *in vivo* with and without PEG

**Figure 4:**
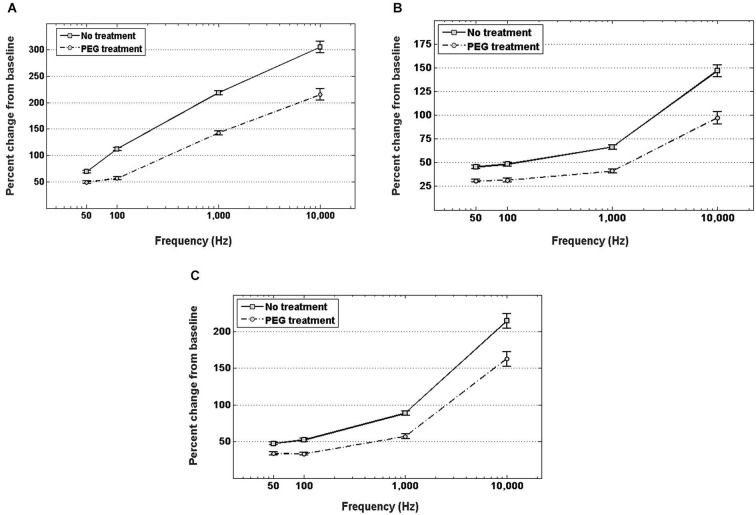
***In vivo* increase in resistance (A), reactance (B), and total impedance (C) from *in vitro* baseline, with and without PEG treatment**. For either treatment condition, a significant increase in impedance (both resistance and reactance) from the *in vitro* baseline is observed at all frequencies (*p* < 0.05). For electrodes with no treatment, the percent increase in impedance (both resistance and reactance) from baseline was significantly higher (*p* < 0.05) than the percent increase with PEG treatment at all frequencies.

Figure 4A shows the percent increase of the real component of the impedance, i.e., resistance, between the *in vitro* baseline and the *in vivo* measurement at four frequency values across the spectrum. In both cases of no treatment and PEG treatment, the resistance exhibited a significant increase when measured *in vivo* compared to the *in vitro* baseline. Insertion into the cortex without PEG treatment, however, resulted in a larger increase from the *in vitro* baseline at all frequencies compared to insertion with PEG treatment. The percent change in resistance was also frequency-dependent. For the no treatment condition, the percent increase from baseline was as follows: 71.8 ± 2.99% at 50 Hz, 89.8 ± 3.77% at 100 Hz, 209.5 ± 9.1% at 1 kHz, and 290.5 ± 13.7% at 10 kHz; while for the PEG treatment condition, the percent increase from baseline was: 44.6 ± 3% at 50 Hz, 58 ± 3.9% at 100 Hz, 149.5 ± 9.3% at 1 kHz, and 223.3 ± 14% at 10 kHz. The percent increase in resistance from baseline for the no treatment condition was significantly different from the percent increase in resistance from baseline for the PEG treatment at all frequencies.

Figure 4B shows the percent increase of the imaginary component of the impedance, i.e., reactance, between the *in vitro* baseline and the *in vivo* measurement at four frequency values across the spectrum. In both cases of PEG treatment or no treatment, the reactance increased significantly when measured *in vivo* compared to the *in vitro* baseline. Insertion into the cortex without PEG treatment, however, resulted in a larger increase in reactance from baseline compared to insertion with PEG treatment. The percent change in reactance was also frequency-dependent. For the no treatment condition, the percent increase from baseline was as follows: 45.4 ± 1.6% at 50 Hz, 48.1 ± 2% at 100 Hz, 66.4 ± 2.3% at 1 kHz, and 146.8 ± 6.2% at 10 kHz; while for the PEG treatment condition, the percent increase from baseline was: 30.8 ± 1.6% at 50 Hz, 31.4 ± 2% at 100 Hz, 41 ± 2.4% at 1 kHz, and 97.1 ± 6.4% at 10 kHz. The percent increase in reactance from baseline for the no treatment condition was significantly different from the percent increase in resistance from baseline for the PEG treatment at all frequencies. The amount of percent change in reactance is lower than the amount of percent change in resistance at each respective frequency. Figure 4C shows the percent increase in total impedance between the *in vitro* baseline and *in vivo* measurement at four frequency values across the spectrum. In both cases of PEG treatment or no treatment, the total impedance increased significantly when measured *in vivo* compared to the *in vitro* baseline. Insertion into the cortex without PEG treatment, however, resulted in a larger increase from baseline compared to insertion with PEG treatment. The percent change in total impedance was also frequency-dependent. For the no treatment condition, the percent increase in total impedance from baseline was as follows: 47.4 ± 1.7% at 50 Hz, 52.2 ± 2.1% at 100 Hz, 88.62 ± 2.9% at 1 kHz, and 214.5 ± 9.8% at 10 kHz; while for the PEG treatment condition, the percent increase in total impedance from baseline was: 33.6 ± 1.6% at 50 Hz, 33.5 ± 2.1% at 100 Hz, 57.1 ± 3% at 1 kHz, and 162 ± 10% at 10 kHz. The percent increase in total impedance from baseline for the no treatment condition was significantly different from the percent increase in total impedance from baseline for the PEG treatment at all frequencies.

## Discussion

### Rationale

The prevalent narrative in the literature suggests that the *in vivo* reactive tissue response is an aggregate of amplified biological processes that begin with device insertion and accompanying trauma. The indwelling implant acts as a sink for various proinflammatory proteins, as well as a substrate for cell attachment (Leung et al., 2008). To the best of the authors’ knowledge, the impedance changes resulting from protein adhesion onto neural microelectrodes have not been previously quantified, nor have the effects of anti-fouling treatments on impedance of neural microelectrodes. Most current efforts towards mitigating the reactive tissue response focus on targeting and quantifying the cellular component of the reactive tissue response. Recent findings, however, indicate that strong cellular responses to implanted microelectrodes do not necessarily correspond to similar changes in electrode impedance (Prasad and Sanchez, 2012). We posit that extracellular components of the reactive tissue response might be more instrumental in altering the electrical properties of implanted electrodes, and thus offer an attractive alternative for quantification and mitigation. The primary objective of this paper was to identify non-cellular components as potential modulators of impedance changes in neural microelectrodes, and to quantify the effects of a specific type of non-cellular component *in vitro* (proteins). The secondary objective was to demonstrate a proof of concept at acute time scales for using PEG applied via a simple dip-coating process to prevent impedance changes caused by non-cellular components both *in vitro* and *in vivo*.

### Explanation of results

For the *in vitro* study with the model protein solution, a three-electrode setup was chosen for its ability to isolate the electrode/solution interface impedance. Changes in impedance measured using this three-electrode setup indicate changes only at the electrode/solution interface (Bard and Faulkner, 2001). Because of the combination of low currents, short timescales, micro-scale working electrodes, and a large area counter, it was assumed that very minimal changes could have occurred at the recording surface. Using this measurement paradigm, our first finding was that significant increases in resistance at all examined frequencies occur immediately upon exposure to a model protein solution with a concentration mimicking *in vivo* concentrations. Furthermore, significant increases in the reactance are observed at higher frequencies, including the physiologically relevant 1 kHz. The aggregate impedance effect is that of a significant increase at frequencies higher than 50 Hz. These observed *in vitro* changes in impedance might not exactly match *in vivo* changes, given the difference in protein composition in the brain, and the presence of additional biomolecules with widely varying degrees of hydrophobicity, such as lipids and polysaccharides (O’Brien and Sampson, 1965; Pease, 1966; Margolis and Margolis, 1974; Norton et al., 1975). This finding of changes in both resistive and capacitive components following protein adsorption challenges current assumptions inherent in prevalent electrical circuit models of the device tissue interface. Such models typically assume that protein adsorption causes purely resistive changes in impedance (Johnson et al., 2005; Otto et al., 2006; Williams et al., 2007). We demonstrate that the exposure of microelectrodes to a protein solution results in both resistive and capacitive changes in impedance, rather than purely resistive changes. These findings suggest a need for the reexamination of assumptions upon which prevalent electrical circuit models for the tissue electrode interface are built. More accurate or representative equivalent circuit models might improve the utility of impedance monitoring to accurately deduce the progression of the reactive tissue response.

Our study further demonstrates that treating electrodes with a PEG film via dip coating prior to immersion in a protein solution negates these increases in impedance at physiologically relevant frequencies. That electrodes pretreated with PEG exhibit slight decreases in reactance supports our working hypothesis that the PEG film forms a hydrated layer close to the electrode surface that prevents proteins from accessing the surface of the electrode, while simultaneously avoiding detrimental effects on charge transfer at the electrode-electrolyte layer.

For the acute *in vivo* study, a two-electrode setup was used due to the difficulty of implementing a three-electrode setup within a surgical setting. The use of a two-electrode setup means that the impedance of the tissue/electrode interface and the impedance of the tissue are lumped into a single impedance source. The expected contribution of the tissue impedance component is confirmed by the considerable increase observed in both the resistance and capacitance following the insertion of the electrode into the rat brain. For this study, we wanted to examine the contribution of non-cellular components to the change in impedance, and therefore chose a short time scale during which no tissue remodeling occurs. Since cellular responses in the brain following electrode implantation are not observed until several hours post implantation (Kozai et al., 2012), our impedance measurement needed to be conducted within that time frame. We chose an arbitrary time point of 5 min post-insertion to minimize the exposure of the animals to unnecessary anesthesia, but it is possible for impedance changes to be time sensitive.

The application of an aqueous solution of a higher molecular weight PEG by dip-coating onto the microelectrode and directly into the craniotomy resulted in a reduction in the magnitude of the increase from the *in vitro* baseline impedance. Because the tissue impedance component is presumed not to change within this short time frame, we can assume that the difference in impedance with PEG treatment compared to the no treatment condition is due primarily to the modulation of the electrode/tissue interface impedance component. In the control craniotomy, we posit that a considerable portion of the immediate impedance increase comes from hydrophobic biomolecules that come into contact with the electrode surface and hinder charge transfer. The application of free-floating high molecular weight PEG appears to confer some degree of protection from the effects of aforementioned biomolecules, and results in lower impedance. With this simple method of applying PEG via dip-coating, there are concerns about the uniformity of coatings between electrodes. While we did not directly quantify the morphology of our dip-coated PEG films, we did attempt to control the deposition process to minimize variability. Since the dip-coating velocity is a major contributing factor to the uniformity of dip-coated films (Scriven, 1988), we controlled our dip-coating velocity by using a micromanipulator. Because we did observe statistically significant changes in impedance in response to our dip coatings, we did not make further attempts to identify and reduce individual contributors to inter-electrode variations.

Much more research needs to be conducted into the use of PEG to understand its potential long-term effects on the functional longevity of implantable intracortical microelectrodes. While prior research in the field has found that conformal microgel PEG coatings that do prevent cellular adhesion *in vitro* do not result in significant improvement to the cellular composition of the chronic electrode tissue interface (Gutowski et al., 2014), the effects of such PEG-containing coatings on the electrical properties of functional electrodes have not been tested. It is possible that significant protective effects using grafted coatings have not been observed due to the use of lower molecular weights. For our proof of concept demonstration for PEG, we used a molecular weight of 4000 Da, which has been shown to be optimal for reducing biofouling in comparison with lower molecular weights (Su et al., 2009). With biopolymers, there are concerns about degradability which might limit the long-term potential for such coatings to modulate the reactive tissue response. PEG, however, is generally not considered easily degradable in physiological conditions, and degradable PEG hydrogels generally need to be engineered with special cleavage sites to facilitate biodegradability (Drury and Mooney, 2003). On the other hand, the effects of long-term presence of large polymeric molecules in the brain should be considered before employing such molecules as neurointegrative coatings.

These complex considerations surrounding such antifouling coatings present an attractive research area. While chemically immobilized coatings are a commonplace choice for neurointegrative coatings, there might be additional merits to weakly attached PEG films. For example, intravenous treatment with higher molecular weight PEG has been shown to improve cellular and behavioral recovery in traumatic brain injury (Koob et al., 2005, 2008; Koob and Borgens, 2006), due to its fusogen properties which allow it to induce membrane sealing of damaged cells and tissue (Shi, 2013). It is possible that these properties of higher molecular weight PEG can be similarly effective in disrupting deposition of proteins and other molecules at the electrode vicinity, in addition to mitigating chronic blood-brain barrier damage, a major factor in chronic device failure (Saxena et al., 2013). Before dip-coated PEG films can be prescribed as a long-term solution for preventing the reactive tissue response and increase the functional longevity of implantable neural microelectrodes, more extensive parametric studies need to be conducted. These studies should consider the molecular weight of the PEG used, in addition to the morphological characteristics of the applied films, such as roughness and thickness, and related properties such as degradation kinetics. The delivery method of PEG films is also an open question. It might be necessary to combine multiple delivery methods; for example, concurrently immobilizing PEG onto the electrode surface and applying free-floating PEG. One potential approach under investigation is the use of thin-film silica sol-gel coatings, which have the potential to incorporate released and immobilized molecules, and do not adversely affect electrical properties (Pierce et al., 2009). Such future experiments with PEG should combine impedance monitoring with histological analysis of the reactive tissue response on a chronic time scale. Our findings provide a first step by highlighting the potential contribution of non-cellular molecular components to impedance changes, and offer a proof of concept for dip-coated PEG films as a potential component within a holistic neurointegrative strategy.

## Conflict of interest statement

The authors declare that the research was conducted in the absence of any commercial or financial relationships that could be construed as a potential conflict of interest.
